# Validity and reliability of the Chinese version of the core extrusion schema-revised for high school students in China

**DOI:** 10.3389/fpsyg.2023.1121197

**Published:** 2023-07-20

**Authors:** Fei Lei, Yue Chen, Shulan Lei, Dacheng Wang, Zhuohong Zhu, Xinying Li, Jing Chen

**Affiliations:** ^1^Key Laboratory of Mental Health, Institute of Psychology, Chinese Academy of Sciences, Beijing, China; ^2^Department of Psychology, University of Chinese Academy of Sciences, Beijing, China; ^3^Department of Psychology and Behavioral Sciences, Zhejiang University, Hangzhou, China; ^4^Wuhan Hanyang District Education Bureau, Wuhan, China

**Keywords:** core extrusion schema, high school students, measurement invariance, psychological assessment, psychological flexibility

## Abstract

**Objective:**

This study evaluated the validity and reliability of the Chinese version of the Core Extrusion Schema-Revised (CES-R) for assessing high school students and measures invariance across gender and grade parameters.

**Methods:**

A sample of 1,334 high school students in Wuhan, China, participated in the study for item analysis, internal consistency tests, and measurement invariance tests of the CES-R. Additionally, 1745 high school students in Zhejiang Province, China. provided data for questionnaire validation.

**Results:**

The results of the confirmatory factor analysis showed that the two-dimensional model fit the data well [chi-squared discrepancy = 113.989; degrees of freedom = 26; Tucker-Lewis index (TLI) = 0.949; comparative fit index (CFI) = 0.963; standardized root mean square residual = 0.072]. The Chinese CES-R scores were positively correlated with both the Adolescent Avoidance and Integration Questionnaire scores (*r* = 0.63, *p* < 0.01) and the Adolescent Social Anxiety Questionnaire scores (*r* = 0.70, *p* < 0.01). The internal consistency coefficient of the questionnaire was 0.94, and the split-half reliability was 0.90. The factor structure invariance, factor loading invariance, and intercept invariance of the Chinese CES-R across gender and grade groups (ΔCFI <0.01, ΔTLI <0.01, ΔRMSEA <0.01) indicated equivalence across gender and grade groups.

**Conclusion:**

The Chinese version of CES-R has good validity and reliability for evaluating high school students and acceptable measurement invariance across genders and grades.

## Introduction

In everyday life, different people react differently when receiving negative comments from others. An established cognitive model known as the core extrusion schema posits that if a person believes that a negative comment about his/her personal characteristics is correct, hiding his/her true personal characteristics can prevent such negative comments (Rodebaugh, 2009). This cognitive pattern avoids the presentation of one’s true self and is one of the phenomena observed in socially anxious people (Young et al., 2003; Rodebaugh, 2009), who show fear of both negative and positive evaluations (Larson and Chastain, 1990; Wei et al., 2005). In fact, regardless of the outcome of self-avoidance, the process of self-non-acceptance can become an internal stressor (Levinson et al., 2015). It was previously shown that avoidance of painful or negative personal information was positively associated with depression and anxiety (Larson and Chastain, 1990; Wei et al., 2005) and increased the risk of psychological discomfort and somatic disorders (Cramer and Barry, 1999). Thus, core extrusion schema may not be specific to socially anxious individuals; rather, it is likely also present in patients with other psychological disorders (Young et al., 2003) or even in healthy individuals, and therefore, the involved schema structures are worthy of further exploration. This dysfunctional schema generally develops early in life as a negative adaptation to the environment (Young et al., 2003). Once an individual forms a core extrusion schema, even if one of their perceptions is in fact inaccurate and distorted, the individual will not easily be able to change this schema to maintain a stable view of self and the world (Young et al., 2003). In particular, high school students are in a critical period of self-concept formation. Failure to develop a correct evaluation and acceptance of self at this time will lead to strong psychological conflicts and may even affect their psychological health in adulthood. Therefore, early identification and intervention of core extrusion schema in adolescents is crucial to promote their future psychological health.

Rodebaugh was one of the first researchers to focus on the core extrusion schema and developed the assessment instrument with the same name: Core Extrusion Schema (CES). He demonstrated the psychometric properties of the CES scale in two preliminary studies using undergraduate sample populations. The CES contains four subscales: (1) Present Rejection (PR), i.e., the current perceived rejection or refusal; (2) Rejection of the True Self (RTS), i.e., the belief that one’s true self will be rejected in social situations; (3) Hidden Self (HS), i.e., trying to hide one’s true self; and (4) Avoid Mistakes (AM), i.e., trying to avoid being scrutinized for social mistakes (Rodebaugh, 2009). Later, Levinson and Rodebaugh noted that although the four factors of the CES fit well together, the PR overlapped with existing social support scales and the AM overlapped with a fear of negativity scale (Rodebaugh, 2009). Therefore, they further revised the 22-item CES scale, retaining only the RTS and HS dimensions, which resulted in a revised 9-item scale. This scale, known as the Core Extrusion Schema-Revised (CES-R) scale (Levinson et al., 2015) has demonstrated good reliability in the U.S. college student population.

To our knowledge, there are no Chinese scales for evaluating core extrusion schema. Compared to Western cultures, Eastern cultures subscribe to more subtle and introverted social norms and tend to behave in a way that avoids attention, conceals their individual characteristics that are distinct from others, and avoids being too prominent among the collective (Zhang et al., 2011). Therefore, an understanding of the core extrusion schema of individuals in the Chinese cultural context would facilitate the cross-cultural study of cognitive schema. Therefore, the goal of the present study was to revise the Chinese version of the CES-R to construct a beneficial tool for understanding the characteristics of cognitive schemas in the Chinese cultural context. We also aimed to develop a valuable tool for Chinese and Western comparisons and cross-cultural studies of core extrusion schema. This study aims to affirm the validity and reliability of the Chinese version of the CES-R for use in the high school student population. Moreover, various research indicates that the overall mental health status of male students outperforms that of female students within the Chinese adolescent population. Furthermore, the mental health status of students varies between different grades (Chen et al., 2020). Consequently, we decided to conduct equivalence tests on the revised questionnaire to confirm its measurement invariance when used for students of various genders and grades.

We examine the applicability of the CES-R in the Chinese culture, and more specifically, in the high school student population, so that it can be adapted in the future for early detection and intervention of core extrusion schema. The results of this study provide new perspectives and measurement tools for the prevention and screening of social anxiety-prone populations.

## Methods

### Study participants

#### Sample 1

We surveyed high school students aged 14–18 in Wuhan, China. Questionnaires were distributed through an online platform. Before the test began, students and their parents were told the purpose of the measurement and asked to read the informed consent form, which stated that they could refuse to answer or quit answering at any time. A total of 1,334 valid questionnaires were collected. The average age of participants was 15.97 (SD = 0.88) years; 588 were male students and 746 were female students; 478 were grade one, 473 were sophomores, and 383 were seniors. For factor analysis, we used SPSS 26.0 to randomly divide Sample 1 (*N* = 1,334) into an exploratory factor analysis (EFA) sample (sample 1.a., *n* = 688) and a confirmatory factor analysis (CFA) sample (sample 1.b., *n* = 646).

#### Sample 2

Students from a high school in Ningbo, China, were selected as another group of subjects, and the relationship between the Chinese version of the CES-R and the adolescent social anxiety questionnaire was examined in this population. Ultimately, 1745 valid questionnaires were collected. Among this population, 934 students were male and 811 were female; their ages ranged from 13 to 19 years, with an average age of 16.08 (SD = 0.91) years.

### Procedure

### Translation of the CES-R

The CES-R was translated into Chinese through a stepwise process. The questionnaire was initially translated into Chinese by two master’s students specializing in psychology. Subsequently, they cross-checked the contents of both translations, addressed the discrepancies, and came to an agreement to shape the first draft of the Chinese version of the questionnaire. Following that, two English graduate students, who have overseas exchange experience and a history of book translations, translated the Chinese draft back into English, then compared with the original English questionnaire, any disparities discussed, and the Chinese questionnaire revised to ensure it faithfully mirrored the original English meaning while remaining clear and understandable in Chinese.

Finally, the corresponding author of this paper and a Chinese psychologist who has lived in the United States for nearly 20 years analyzed the differences between the English questionnaire and the revised Chinese questionnaire. They discussed the results and determined the optimal presentation of the Chinese version to make the language clearer, easier to understand, and more consistent with Chinese expression habits, while ensuring that the original intention of the compiler was reflected.

The translated questionnaire was first administered to a group of 30 high school students to confirm that the meaning of each question could be accurately understood. Several of the participants had no opinion on the question formulation but consistently reported that the original questionnaire (a Likert 10 scale of 0–9) included too many choices, and they found it cumbersome and difficult to choose an answer. It has been shown that scales with multiple potential answers require more time for participants to respond than scales with fewer answer choices (Preston and Colman, 2000). However, the number of answer options has little influence on the reliability of the scale (Matell and Jacoby, 1972; Howard and Margaret, 1975). Taherdoost (2019) compared scales with different ratings and concluded that a 7-point scale and a 6-point scale are optimal. The present study considered the work of two psychologists and changed the scoring of the Chinese version of the CES-R to a 6-point scale, where 1–6 corresponded to answers ranging from “not at all” to “very much.” After this modification, the questionnaire was administered on a large scale.

This study utilized a web-based platform for the questionnaire’s editing and distribution. During the editing process, all questionnaire items were made compulsory, precluding submission if any were left unanswered.

### Instruments

Avoidance and Fusion Questionnaire for Youth (AFQ-Y8) (Greco et al., 2008):

This questionnaire assesses the psychological flexibility of adolescents. Individuals with low psychological flexibility are more inclined to adopt avoidance-based and ineffective behaviors, which lead them further and further from their ideal life. The questionnaire has eight items and is scored on a 5-point scale from 1 (not at all) to 5 (fully), with higher scores indicating more severe psychological rigidity and lower levels of psychological flexibility. The Chinese version of the AFQ-Y8 has been confirmed to have good reliability and validity (Chen et al., 2019), and in the present study, this questionnaire was administered to Sample 1 participants (Cronbach *α* coefficient = 0.84).

Chinese version of the Brief Form of the Social Phobia and Anxiety Inventory for adolescent (SPAI-B) (Liu et al., 2017):

This questionnaire assesses the level of social anxiety in adolescents based on 16 items scored on a 5-point scale from 1 (never occurs) to 5 (always occurs). The first 14 items evaluate behavior, the 15th item evaluates cognition, and the 16th item evaluates somatization symptoms, with higher scores indicating more prominent social anxiety symptoms. Liu et al. (2017) examined the reliability and validity of this questionnaire in a Chinese adolescent population. The scale was administered to Sample 2 participants in the present study (Cronbach *α* coefficient = 0.96).

### Statistical analyses

Common method biases were analyzed by controlling for the effects of an unmeasured latent methods factor (ULMC) (Zhou and Long, 2004). Moreover, EFA was conducted to explore the underlying factor structure of the scale, and CFA was conducted to assess the fitting of model data. We used parallel analysis and principal component analysis with varimax rotation to perform EFA. The goodness of CFA fit should be evaluated against well-established criteria as follows: a standardized root mean square residual (SRMR) below 0.05, a Comparative Fit Index (CFI) and Tucker-Lewis index (TLI) greater than 0.90, a parsimony-adjusted comparative fit index above 0.50, and a root mean square error of approximation (RMSEA) below 0.05 (Wen et al., 2004). We used Pearson’s correlation principle to examine the correlations among all variables and items. The Maximum Likelihood Method (MLM) was used to compare the configural invariance (Model 1), metric invariance (weak invariance, Model 2), scalar invariance (strong invariance, Model 3), and error variance invariance (strict invariance, Model 4) models to determine whether the Chinese CES-R scale was equally applicable across gender and grade levels. Because the chi-square test (*χ*^2^) is oversensitive in the assessment of invariance in large samples (*N* > 300)(Chen, 2007), the Chinese version of the CES-R will be evaluated using ΔCFI and ΔTLI and Bayesian Information Criterion indicators (BIC) across gender and age measurement invariance tests (Wang et al., 2012; He et al., 2017). The measurement invariance model is acceptable when ΔCFI and ΔTLI are less than 0.01, and the BIC value decreases when the models are compared (Cheung and Rensvold, 1999, 2002; Sala et al., 2012). The SPSS 26.0 software was used to calculate reliability, relevance, and EFA, while Mplus 8.0 was used to calculate common method bias and CFA.

## Results

### Common method biases test

The data for this study were all obtained from self-reported questionnaires, making it necessary to test for common method bias. An EFA was performed for all entry scores of the CES-R, AFQ-Y8 questionnaire for sample 1. There were two factors based on the principle of eigenvalues >1. Validating factor analysis was then performed using sample b. The fit of the one-factor model was tested, and the results indicated a poor fit (chi-squared discrepancy [*χ*^2^] = 1524.335; degrees of freedom [*df*] = 119; CFI = 0.844; TLI = 0.821; RMSEA (90% CI) = 0.094 (0.09, 0.098); SRMR = 0.075). Then, the two-factor model fit was obtained (*χ*^2^ = 726.876; *df* = 118; CFI = 0.932; TLI = 0.922; RMSEA = 0.062; SRMR = 0.041), and a method factor was added to develop a three-factor model (ΔCFI = 0.02; ΔTLI = 0.01; ΔRMSEA = 0.01). However, the model fit index did not improve significantly (ΔCFI <0.1; ΔTLI <0.1; ΔRMSEA <0.05)，indicating that no significant common method bias exists (Hau et al., 2004).

### Item analysis

In this study, the average CES score for the sample was 21.53, with a standard deviation of 10.96 and a score range from 9 to 54. Sample 1 (*N* = 1,334) was sorted by total CES-R scores from highest to lowest, and participants were divided into high and low subgroups according to the upper and lower 27% criteria. An independent sample t-test was conducted between the high and low subgroups for each entry score in the scale, and the results showed that the difference in scores between the high and low subgroups on each entry reached a statistically significant level (Cohen’s *d* = 2.06–2.90, *p* < 0.01), indicating that each entry was discriminatory. Pearson correlation coefficients between entries were also calculated, and the results showed that all entries were positively correlated with one another (*r* = 0.48–0.75, *p* < 0.001). There were no pairs of entries with *r* > 0.80, indicating that the contents of the entries were interrelated and did not overlap (Wong et al., 2017). The scores for each question and their correlations with the total score of the scale are presented in Table 1. Pearson correlation coefficients indicated that each entry score was positively correlated with the total questionnaire score (*r* = 0.75–0.86, *p* < 0.001), and the homogeneity of the questions and scales was high. These results indicated that each entry had good consistency and supported the retention of all entries.

**Table 1:** Scores for each item and their correlations with the total score of the scale (*N* = 1,334).

Items	Mean	SD	Correlation between the item and the total score
1 If people really knew me, they would not like me as much. Translation:人们如果真的了解我，就不会那么喜欢我了。	2.51	1.60	0.82^**^
2 I rarely act “like myself” around others. Translation:在别人面前我很少表现得“像我自己”。	2.56	1.54	0.81^**^
3 Many people who think they are close to me do not actually know me very well. Translation:很多认为和我亲近的人其实并不十分了解我。	3.05	1.64	0.75^**^
4 I put on an act around most people. Translation:在多数人面前我是装模作样或“戴着面具”的。	2.33	1.48	0.83^**^
5 Most people do not really like the kind of person I really am. Translation:大多数人实际上不喜欢我真实所是的那种人。	2.16	1.42	0.86^**^
6 If I said what I really think, people would probably reject me. Translation:如果我说出我的真实想法，人们可能会排斥或拒绝我。	2.38	1.47	0.81^**^
7 I generally put up a front while interacting with people. Translation:与人交往时，我通常隐藏或掩饰自己。	2.00	1.32	0.77^**^
8 I’m afraid that people will realize what I’m really like. Translation:我害怕别人会意识到我的真实状态。	2.33	1.51	0.83^**^
9 If I opened up to people, it would go badly. Translation:如果我向人们敞开心扉，情况可能会变糟。	2.23	1.52	0.82^**^

**p < 0.01.

### Structural validity

### EFA

**Figure 1:**
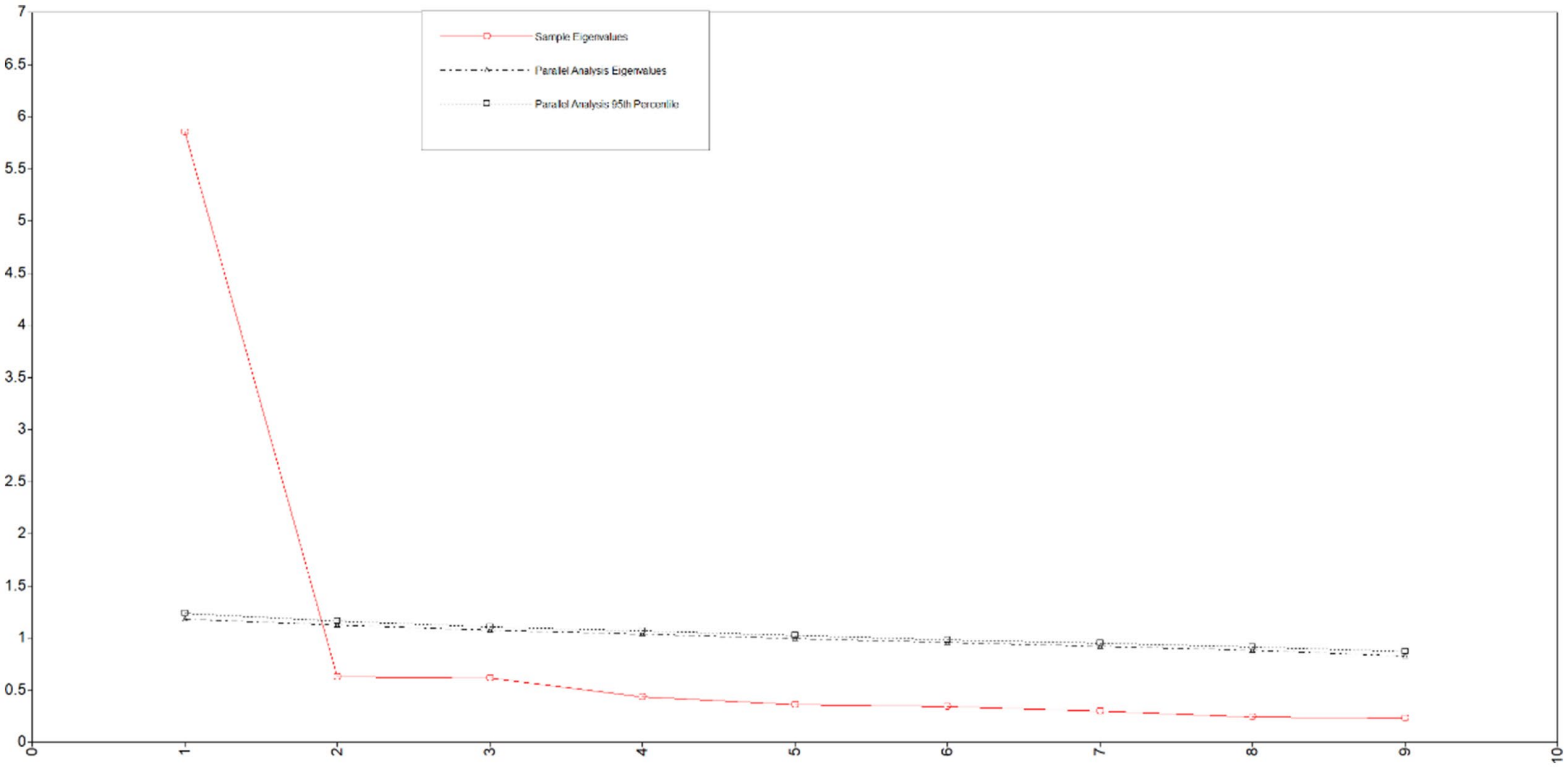
The Chinese version of CES-R parallel analysis Scree Plot (*n* = 688).

The EFA method revealed the potential latent variables of the Chinese version of the CES-R. The results of the parallel analysis using sample 1.a. are shown in Figure 1. The eigenvalue curves of the real and simulated data intersected between the first and second factors. The eigenvalue of the second factor of the real data (0.668) is smaller than the average eigenvalue of the second factor of the random data (1.118), indicating that the variation explained by the first factor is significantly different from the variation caused by random error, suggesting that it is appropriate to extract one factor. The Kaiser-Meyer-Olkin Index (0.94) and Bartlett sphericity test (*χ*^2^ = 4609.18; *df* = 36; *p* < 0.001) indicated that the data were adequate to perform factor analysis. The items with factor loading less than 0.45, cross-loadings within 0.20, or communality less than 0.40 were deleted. The limit is to extract one factor by principal component analysis with varimax rotation. No items were deleted. The factors explained 67.2% of the total variance. The factor loadings of each item ranged from 0.80 to 0.87, and the variance of the common factor ranged from 0.49 to 0.76 (Table 2).

**Table 2:** Items and factor loadings for the CES-R (*n* = 688).

Items	Factor loading	Communalities
Item 1	0.820	0.672
Item 2	0.831	0.690
Item 3	0.699	0.488
Item 4	0.859	0.738
Item 5	0.870	0.756
Item 6	0.826	0.682
Item 7	0.799	0.638
Item 8	0.836	0.699
Item 9	0.828	0.685

### CFA

**Figure 2:**
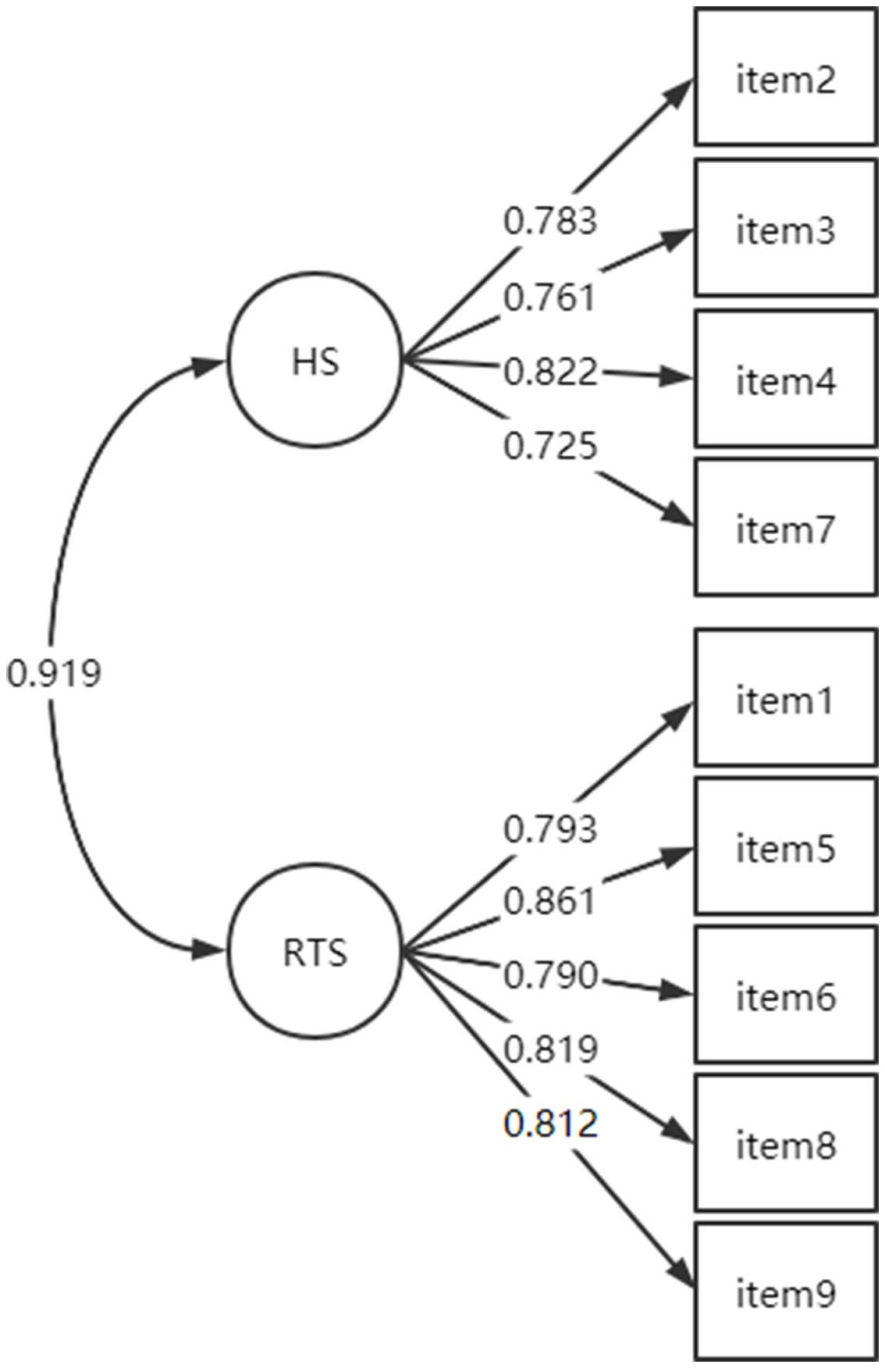
Measurement model for two-factor model (*n* = 646).

CFA was conducted using data from sample 1.b. (*n* = 646) to compare the fit indices of the two competing models: the single-factor model obtained from the CFA of this study and the two-factor model of the original scale. The results are compiled in Table 3, which indicates that the two-factor model fits better with the experimental data. The CFA results support the two-factor structure of the questionnaire (factors RTS and HS). The result of the two-factor model is shown in Figure 2. Subsequently, a chi-square difference test was used to compare the two models. Since the MLM method was employed, the calculated chi-square values required Satorra-Bentler correction (S-B*χ*^2^), as their distribution diverges from the normal chi-square distribution. Thus, they needed recalculating with the formula: ML*χ*^2^ = correction factor * S-B*χ*^2^. The results indicated that *p* = 0.000, demonstrating a significant difference between the two models. Further support for the two-factor structural model of the questionnaire was gleaned from the fit index information. Table 4 presents the results of the model comparison.

**Table 3:** Goodness-of-fit indices for the Chinese version of the CES-R (*n* = 646).

	*χ* ^2^	*df*	CFI	TLI	SRMR	RMSEA
Single factor model	151.233	27	0.948	0.931	0.036	0.084
Two-factor model	113.989	26	0.963	0.949	0.030	0.072

**Table 4:** The results of the model comparison (*n* = 646).

Model	S-B*χ*^2^	df	correction factor	ML*χ*^2^	ΔML*χ*^2^	Δdf	*p*
M2	113.989	26	1.787	203.67			
M1	151.233	27	1.798	271.96	68.294	1	0.000

Note: ML*χ*^2^ = correction factor * S-B*χ*^2^.

### Correlations between Chinese CES-R and other measures

Table 5 shows the correlation coefficients between the scores of each subscale in the Chinese version of the CES-R and those on the AFQ-Y8 (Sample 1) and SPI-B (Sample 2). The results indicate that the Chinese version of CES-R positively correlated with both AFQ-Y8 and SPAI-B.

**Table 5:** Correlations between the CES-R and other variables.

Measure	Sample 1 (*N* = 1,334)			Sample 2 (*N* = 1745)		
	AFQ-Y8	CES-R total score	HS	SPAI-B	CES-R total score	HS
CES-R total score	0.63**			0.74**		
HS	0.58**	0.941**		0.70**	0.96**	
RTS	0.61**	0.964**	0.82**	0.73**	0.97**	0.88**

**p < 0.01.

### Consistency and reliability

We then calculated Cronbach’s *α* coefficient for the total scale and each subscale of the Chinese version of the CES-R and obtained acceptable results. The results of each subscale shown in Table 6 indicate that the scale has good reliability.

**Table 6:** Cronbach’s *α* coefficient for each subscale of the Chinese version of the CES-R and for the Chinese version of the CES-R as a whole.

	CES-R total score	HS	RTS
Cronbach’s *α*	0.94	0.86	0.91

### Measurement invariance test

The scale was examined for measurement invariance across grade and gender in Sample 1 to demonstrate the appropriateness of the scale in terms of these parameters. Comparing the morphological, weak, strong, and strict equivalence models revealed that the CFI, TLI, and RMSEA values of each model met certain psychometric criteria, ΔCFI and ΔTLI were less than 0.01 between models, and the value of BIC decreases in descending order (Table 7). It can be inferred that the CES-R scale exhibits measurement invariance across gender and grade levels of high school students.

**Table 7:** Validated factor analysis of multi-group comparison nested model fit indices.

Measurement invariance	Model	*χ* ^2^	*df*	TLI	CFI	RMSEA	SRMR	BIC	ΔTLI	ΔCFI
Gender	Model 1	366.347	129	0.956	0.963	0.061	0.029	27067.674	–	–
	Model 2	369.667	136	0.959	0.964	0.059	0.03	27027.492	0.003	0.001
	Model 3	378.903	145	0.962	0.964	0.057	0.033	26980.796	0.003	0
	Model 4	405.448	154	0.961	0.961	0.057	0.036	26951.410	−0.001	−0.003
Grade	Model 1	649.753	309	0.932	0.941	0.061	0.037	24536.801	–	–
	Model 2	662.764	323	0.936	0.941	0.059	0.04	24469.959	0.004	0
	Model 3	675.311	341	0.940	0.942	0.057	0.044	24379.838	0.004	0.001
	Model 4	694.796	359	0.943	0.941	0.056	0.044	24296.655	0.003	−0.001

## Discussion

The core extrusion schema is a relatively new concept that is closely associated with social anxiety. This study revised the Chinese version of the CES-R and validated its reliability in two groups of high school students. The item-item correlations and total questionnaire correlations involving the nine items of the scale met psychometric criteria and supported the retention of all items. The CFA results indicated that the Chinese CES-R scale adopted the same two-dimensional structure as the original scale. The reliability analysis indicated good internal consistency of the scale and high homogeneity among entries. This study also showed that the Chinese CES-R scale has measurement invariance characteristics across gender and grade levels, meaning its scores can be compared across gender and grade levels. Therefore, it can be concluded that the Chinese CES-R scale is a valid instrument for measuring core non-acceptance iconography in high school students.

EFA results showed extraction of one factor, while CFA showed better fitting results for the same two-factor model as the original English scale. The results of the validation factor analysis in this study showed that the Chinese version of the CES-R scale has the same two dimensions (hidden self and rejection of the true self) as the original scale in English. Results from studies of college students in collectivist cultures, e.g., Japan and Korea, indicate that college students in these countries identify more with social norms of reticence, subtlety, and introversion, and tend to behave in a more attention-averse manner (Schreier et al., 2010). The Chinese, who also have a collectivist culture, emphasize seeking commonality and harmony, subordinating themselves to the collective, obscuring their individual characteristics that are different from others, and avoiding being too prominent among the collective (Zhang et al., 2011). This maneuver allows RTS and HS to develop and influence each other as the youth matures. However, there may be differences in the developmental order and progressive structure of HS and RTS for adolescents in China and Western countries due to cultural influences; therefore, a distinction between the two is necessary for further research. The concept of CES also provides theoretical support for the two-factor model. Moreover, the results of the CFA showed that the model with two factors had a better fit. Therefore, we decided to take the results of the CFA and concluded that the scale has two dimensions, RTS and HS.

The original CES-R scale has been used to assess social anxiety in adults. The results of the present study showed that the Chinese version of the CES-R score was correlated with the SPI-B, indicating a correlation between the core extrusion schema and social anxiety in Chinese adolescents. The stronger the core extrusion schema, the more an individual tends to believe that they are not acceptable; this makes them afraid of being “discovered” when socializing, which induces feelings of stress and anxiety. Therefore, the Chinese version of the CES-R scale can be used as an indicator when screening social anxiety in high school students and implementing preventive factors. Besides, the results of the present study showed a moderate positive correlation between Chinese CES-R scores and AFQ-Y8 scores. This result suggests that CES can be understood from an Acceptance and Commitment Therapy (ACT) perspective. Individuals with core extrusion schema automatically confuse thought content with “facts,” treat thoughts and feelings as external facts, activate emotional experiences and behavioral responses associated with specific schema, and behave in ways that are heavily influenced by thought content, which is a manifestation consistent with the definition of cognitive fusion in the pathological model of ACT (Zhang et al., 2014). The role and pathways of the core extrusion schema in ACT interventions for social anxiety need to be further investigated, and the CES-R questionnaire provides a tool for future research in this area.

### Limitations

In conclusion, the validity and reliability of the Chinese CES-R scale for evaluating high school students meets the psychometric requirements, and it is therefore a valid tool for measuring the core extrusion schema of high school students in China. Unfortunately, however, the present study represents the first attempt to explore the structure and measurement instruments of the core extrusion schema in the Chinese cultural context using the Chinese CES-R scale. Moreover, the data were collected anonymously and could not be analyzed for retest reliability, although this will be addressed in future studies. In addition, the sample populations in this study were all high school students, and thus, a more broadly representative sampling of different ages in China is needed to validate the scale’s wider applicability for measuring psychometric indicators in the future. The sample assessed in this study was a non-psychological disorder group, so future research should explore the influence of the core extrusion schema and its mechanisms of action on individuals with social anxiety or other psychological behavioral problems in China. Finally, this study collected cross-sectional data; future longitudinal studies could be conducted to further explore the formation and development of the schema and its related influences.

## Data availability statement

The raw data supporting the conclusions of this article will be made available by the authors, without undue reservation.

## Ethics statement

Ethical review and approval was not required for the study on human participants in accordance with the local legislation and institutional requirements. Written informed consent to participate in this study was provided by the participants’ legal guardian/next of kin.

## Author contributions

FL and YC made main contribution to this study, including come up with ideal, analyzed the data, wrote, and revised the manuscript. SL was in charge of collecting the data. ZZ and XL provided data analysis strategies of this study. JC provided suggestions on revision and confirmed the final version to be published. All authors contributed to the article and approved the submitted version.

## Conflict of interest

The authors declare that the research was conducted in the absence of any commercial or financial relationships that could be construed as a potential conflict of interest.

## Publisher’s note

All claims expressed in this article are solely those of the authors and do not necessarily represent those of their affiliated organizations, or those of the publisher, the editors and the reviewers. Any product that may be evaluated in this article, or claim that may be made by its manufacturer, is not guaranteed or endorsed by the publisher.
